# Practical use of visual medial temporal lobe atrophy cut-off scores in Alzheimer’s disease: Validation in a large memory clinic population

**DOI:** 10.1007/s00330-016-4726-3

**Published:** 2017-01-12

**Authors:** Jules J. Claus, Salka S. Staekenborg, Dana C. Holl, Jelmen J. Roorda, Jacqueline Schuur, Pieter Koster, Caroline E. M. Tielkes, Philip Scheltens

**Affiliations:** 1 Department of Neurology, Tergooi Hospital, Blaricum, The Netherlands; 2 Department of Neurology, Alzheimer Center, VU University Medical Center, de Boelelaan 1118, 1081 HZ Amsterdam, The Netherlands; 3 Department of Geriatrics, Tergooi Hospital, Blaricum, The Netherlands; 4 Department of Radiology, Tergooi Hospital, Blaricum, The Netherlands; 5 Department of Medical Psychology, Tergooi Hospital, Blaricum, The Netherlands

**Keywords:** Alzheimer’s disease, Temporal lobe, Diagnostic imaging, Computed tomography, Clinical practice

## Abstract

**Objective:**

To provide age-specific medial temporal lobe atrophy (MTA) cut-off scores for routine clinical practice as marker for Alzheimer’s disease (AD).

**Methods:**

Patients with AD (n = 832, mean age 81.8 years) were compared with patients with subjective cognitive impairment (n = 333, mean age 71.8 years) in a large single-centre memory clinic. Mean of right and left MTA scores was determined with visual rating (Scheltens scale) using CT (0, no atrophy to 4, severe atrophy). Relationships between age and MTA scores were analysed with regression analysis. For various MTA cut-off scores, decade-specific sensitivity and specificity and area under the curve (AUC) values, computed with receiver operator characteristic curves, were determined.

**Results:**

MTA strongly increased with age in both groups to a similar degree. Optimal MTA cut-off values for the age ranges <65, 65–74, 75–84 and ≥85 were: ≥1.0, ≥1.5, ≥ 2.0 and ≥2.0. Corresponding values of sensitivity and specificity were 83.3% and 86.4%; 73.7% and 84.6%; 73.7% and 76.2%; and 84.0% and 62.5%.

**Conclusion:**

From this large unique memory clinic cohort we suggest decade-specific MTA cut-off scores for clinical use. After age 85 years, however, the practical usefulness of the MTA cut-off is limited.

***Key Points*:**

• *We suggest decade-specific MTA cut-off scores for AD.*

• *MTA cut-off after the age of 85 years has limited use.*

• *CT is feasible and accurate for visual MTA rating.*

## Introduction

Medial temporal lobe atrophy (MTA) is considered as a biomarker for Alzheimer’s disease (AD) [1–6] and visual MTA ratings are available for clinical use [7]. There is debate as to what cut-off scores should be used in clinical practice to optimally differentiate AD from controls without dementia [8] or with other types of dementia [9, 10]. One of the main problems is that MTA increases with age and cut-off scores should be adjusted for age [11, 12]. However, few studies are available addressing this issue.

MTA cut-off scores were investigated in two recent studies that suggest increasing these scores with 0.5 per decade in elderly patients [12, 13]. If evaluated in clinical settings, these cut-off values might prove to be an aid in the diagnostic evaluation of AD. This testing is necessary since AD patients in these studies were not representative for those in a general hospital memory clinic and no data are available on computed tomography (CT). Furthermore, relatively few patients over 80 years of age were included, and specifically in these elderly patients MTA differentiation of AD and a reference group may be problematic due to the age effect [14].

Therefore, we test these decade-specific MTA cut-off scores in a single-centre memory clinic population including a large sample of patients over 80 years of age and assess the use of MTA in clinical practice. We defined decade-specific MTA cut-off values that best discriminate between AD and subjective cognitive impairment (SCI) using CT scans with visual rating of MTA.

## Methods

### Subjects

Patients included in this study were referred because of cognitive complaints to the memory clinic at Tergooi Hospital, a general hospital in Hilversum and Blaricum, The Netherlands. Since April 2009, we use a standard protocol for diagnostic assessment, based on the healthcare pathway of the VUmc Alzheimer Center with organization in a one-stop shop modality [15]. Each patient received the same diagnostic work-up in one day, resulting in a total of close to 350 patients per year. This resulted in a consecutive series of 2,000 patients in a period of 6 years, from April 2009 to April 2015 (for a summary of the study population and procedures see Claus et al. [16]). All patients diagnosed with AD (832) or SCI (333) were included in the current study.

### Clinical diagnostic procedures

All patients completed the following diagnostic evaluation: (1) a full medical and neurological examination including history-taking by a neurologist or geriatrician, (2) assessment of vital functions, (3) cognitive screening with a CAMCOG test part of the CAMDEX, (4) standard electrocardiogram, (5) laboratory tests and (6) informant-based history and assessment of needs by a specialized nurse including admission of the Geriatric Depression Scale (GDS) and assessments of the Instrumental Activities of Daily Living Scale. The clinical diagnosis was made in a consensus meeting attended by the neurologist, geriatrician, neuropsychologist and a specialized nurse. AD diagnosis was made using the current standard clinical diagnostic criteria for AD [3]. MTA rating was not used in the diagnostic procedure.

If patients scored normally on all tests and no other diagnosis could be made, patients were considered as having SCI. These patients were used as the reference group.

### Computed tomography (CT) protocol

CT scanning of the brain was performed using a 64-detector row CT with Siemens Somatom definition AS 64-slice scanner according to a CT brain protocol for the memory clinic (260 mAs, 120 kV, 64 * 0.6 mm collimation, pitch of 0.55, WC/WW = 40/80, CARE kV = on (dose optimation slider on non-contrast)). Oblique coronal, sagittal and transverse reconstructions were made with bone-window 1.5-mm slices, axial slices of 5.0 mm and oblique coronal slices of 3.0 mm, modified from the protocol described by Wattjes et al. [17]. All CT scans were reviewed by a radiologist in the routine procedure of patients presenting to an outpatient memory clinic to exclude any other underlying disease that could explain cognitive decline. The report of the radiologist was not used in a structured (MTA) or unstructured way for the diagnosis of AD at the multidisciplinary meeting.

CT scans were visually assessed for MTA by applying the 5-point rating scale from 0 (no atrophy) to 4 (maximum atrophy) as proposed by Scheltens et al. [7]. The right and left hemisphere were rated separately, the MTA score being the average of these two values. This visual assessment was made in a consensus meeting by a neurologist and geriatrician. Beforehand, these specialists had received instructions on how to perform an MTA rating according to the original study by Scheltens et al. [7], by regular visits of Prof. Scheltens to Tergooi Hospital.

Intra-rater and inter-rater variability was assessed using a randomly selected set of 20 CTs that was visually rated for right and left MTA, blinded for age and diagnosis, by two combinations of the neurologist and geriatrician as employed for consensus rating in our daily memory clinic practice, as well as by an experienced neuroradiologist. This allowed comparison between two teams of neurologist and geriatrician and of these teams with the radiologist. The same set was rated 1 week later by the same persons allowing intra-rater variability testing.

### Statistical analysis

We used SPSS version 22.0. Baseline characteristics were analysed with one-way ANOVA or with chi-square tests when appropriate. The relationship between age and MTA scores was assessed with multiple linear regression analysis, with adjustment for gender and level of education in AD patients and SCI patients separately. Interaction between age and diagnosis in relation to MTA was investigated with two-way independent ANOVA (general linear model). In other words, we assessed whether the slopes of the regression lines were significantly different between groups. In addition, the relationship between age and MTA was determined separately in those aged under and over 80 years to determine specific age-related effects on MTA scores. Eighty years was chosen as the age for analysis to define elderly AD patients since in the literature few data are available on the relationship between age and MTA above 80 years of age.

The sensitivity and specificity of different MTA cut-off scores were computed stratified in four decade-specific groups: <65, 65–74, 75–84, ≥85 years. Statistical analyses were performed using chi-square tests. Diagnostic performance was further investigated with receiver operator characteristic (ROC) curves with corresponding areas under the curve (AUC) and 95% confidence intervals. Optimal combinations of sensitivity and specificity were defined according to the highest AUC value, unless a more favourable combination of sensitivity and specificity was present for clinical purposes, i.e. higher specificity to avoid false positives, with a comparable AUC value.

Intra-rater and inter-rater variability were computed using the intraclass correlation coefficient (ICC) with a two-way mixed absolute agreement and single-measures design. We calculated the following ICC values with 95% confidence interval (CI) for mean of right and left MTA score: (1) intra-rater reliability for first and second MTA rating for the two teams of neurologist and geriatrician and for the neuroradiologist, (2) inter-rater reliability between the first and second team of neurologist and geriatrician, and (3) inter-rater reliability between either the team of neurologist and geriatrician and the neuroradiologist.

## Results

**Fig. 1:**
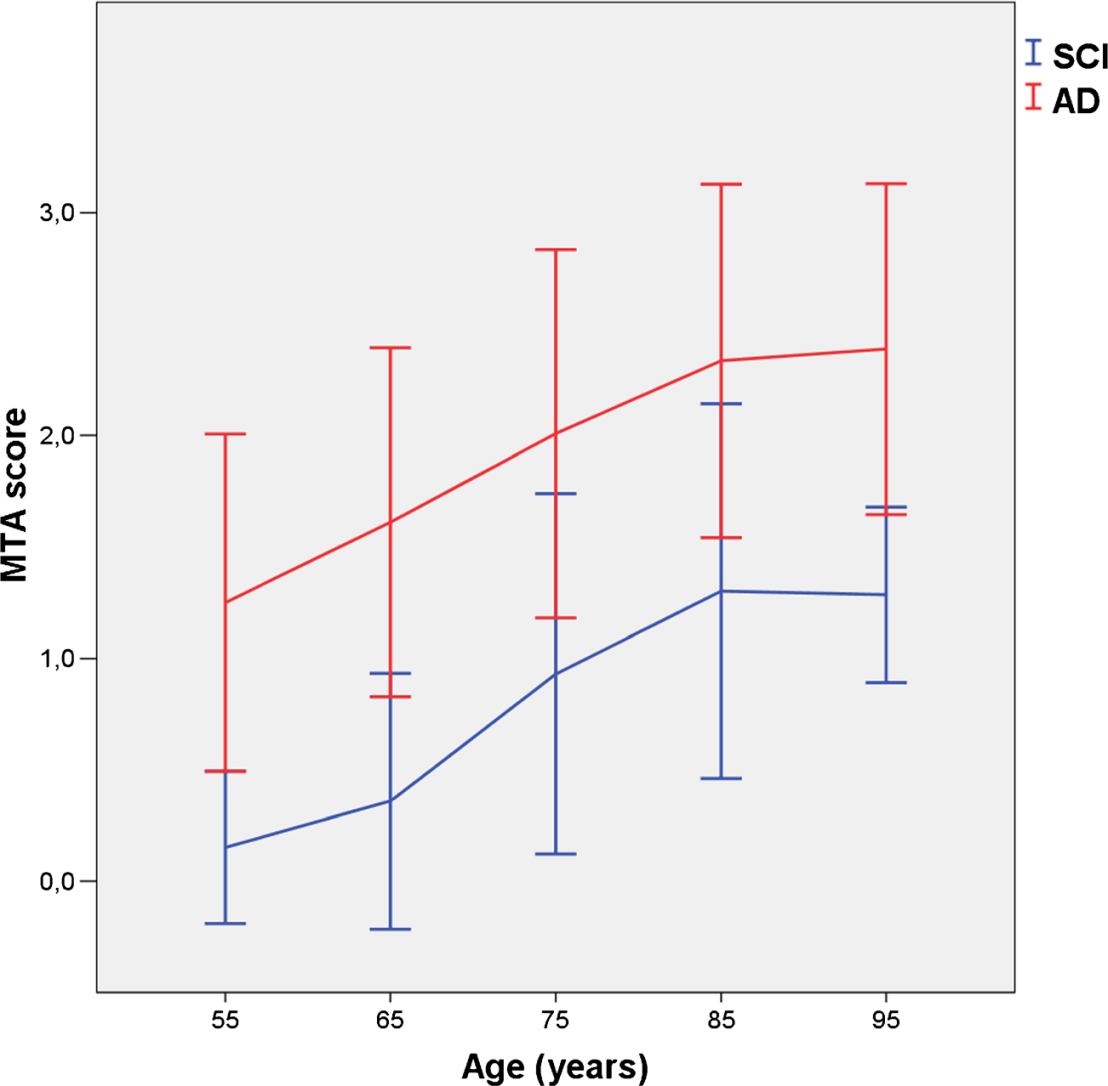
Medial temporal atrophy (MTA) scores (visual rating, Scheltens scale) on computed tomography in patients with subjective cognitive impairment (SCI) and Alzheimer’s disease (AD) in relation to age. The x-axis shows age (years), the y-axis shows mean of right and left MTA scores (± 1 standard deviation). Age correlated significantly with MTA in both SCI and AD patients, adjusted for gender and level of education. The effect of age on MTA was similar in both groups and was stronger before 80 years than after 80 years

The clinical characteristics and frequencies of MTA scores are shown in Table 1. The mean age of the total population was 78.9 years (range 45–96 years). SCI patients had a lower percentage of female persons than AD patients (p < 0.01), were significantly younger (p < 0.001), had a higher level of education (p < 0.001) and a higher MMSE score (p < 0.001), and lower mean MTA than AD patients (Table 1). Regression analysis showed statistically significant relationships between age and MTA, adjusted for gender and education for both SCI and AD patients in separate analyses with regression coefficients of 0.043 ± 0.004 (p < 0.001) and 0.036 ± 0.004 (p < 0.001), respectively. Interaction analysis showed no differential effect of diagnosis on the relationship between age and MTA (p = 0.26). Thus, the effect of age on MTA was similar in SCI and AD patients. These relationships were also computed in those under 80 years of age and in patients of 80 years of age and above. Regression coefficients for SCI patients were 0.040 ± 0.005 (p < 0.001) and 0.027 ± 0.027 (p = 0.34), respectively, and for AD patients 0.046 ± 0.010 (p < 0.001) and 0.020 ± 0.010 (p < 0.05), respectively. Regression coefficient values are almost halved in patients over 80 years of age when compared with those under, and the relationship between age and MTA is no longer significant above the age of 80 years in SCI patients, possibly explained by the lower number of patients in this age group. It thus appears that the age effect is stronger below 80 hyears than in 80 years and above but to a similar degree in SCI and AD. See Fig. 1 for MTA decade-specific values in relation to age for SCI and AD separately.

**Table 1:** Demographic characteristics and distribution of medial temporal atrophy scores

	All participants	SCI	AD	*p* values
No.	1165	333	832	
Male/female	440/725	146/187	294/538	<0.01
Age, y, mean (SD)	78.9 ± 9.1	71.8 ± 10.2	81.8 ± 6.9	<0.001
Education (Verhage)	4.3 ± 1.5	4.7 ± 1.4	4.2 ± 1.5	<0.001
MMSE	20.6 ± 6.1	27.2 ± 2.3	18.0 ± 5.0	<0.001
MTA	1.8 ± 1.1	0.7 ± (0.8	2.2 ± 0.8	<0.001
MTA score 0, n (%)	161 (13.9%)	148 (44.4%)	13 (1.6%)	
0,5	26 (2.2%)	20 (6.0%)	6 (0.7%)	
1	210 (18.1%)	98 (29.4%)	112 (13.6%)	
1.5	95 (8.2%)	17 (5.1%)	78 (9.5%)	
2	293 (25.3%)	35 (10.5%)	258 (31.3%)	
2.5	126 (10.9%)	7 (2.1%)	119 (14.4%)	
3	181 (15.5%)	7 (2.1%)	174 (21.1%)	
3.5	38 (3.3%)	1 (0.3%)	37 (4.5%)	
4	28 (2.4%)	0 (0%)	28 (3.4%)	

Comparisons of variables were made with one-way ANOVA or with Chi-square test (gender)

*SCI* subjective cognitive impairment , *AD* Alzheimer’s disease , *MMSE* Mini-Mental State Examination , *MTA* medial temporal atrophy, *Verhage* years of education following primary school

**Fig. 2:**
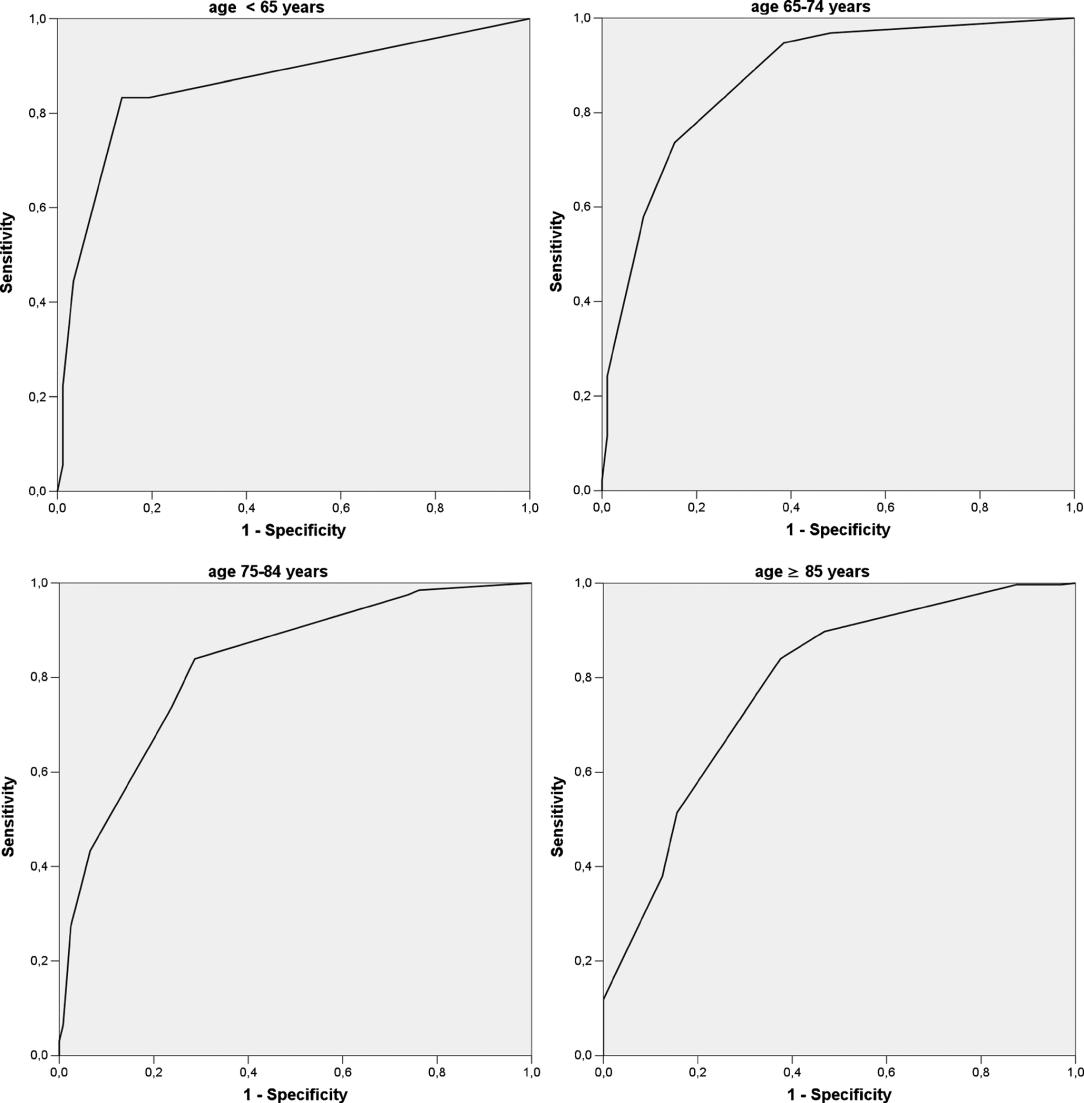
Diagnostic performance of the visual medial temporal atrophy (MTA) rating scale (Scheltens) with receiver operator characteristic curves comparing patients with subjective cognitive impairment and Alzheimer’s disease per age decade. Optimal cut-off values are: ≥1.0 for <65, ≥1.5 for 65–74, ≥ 2.0 for 75–84, and ≥2.0 for ≥85 years

The sensitivity and specificity of different MTA decade-specific cut-off scores are shown in Table 2 and ROC curves are shown in Fig. 2 (total number of patients 1,158; seven scans were missing). Optimal decade-specific MTA cut-off scores were the following: <65 years, MTA cut-off value ≥1.0 with specificity of 86.4%, sensitivity of 83.3% at the highest AUC value of 84,8; 65–74 years, MTA cut-off value ≥1.5 with specificity of 84.6% and a sensitivity of 73.7% at the highest AUC value of 79,1; 75–84 years, MTA cut-off value ≥2.0 with specificity of 76.2% and sensitivity of 73.7% at an AUC value of 75.0; this value is comparable to the highest value of 77.6, therefore in this instance we chose for the cut-off of ≥2.0 instead of ≥1.5 because the higher specificity and somewhat lower sensitivity are preferred for clinical use to reduce the number of false positives; ≥85 years MTA cut-off value ≥2.0 with specificity of 62.5% and a sensitivity of 84.0% at the highest AUC value of 73.3, although specificity is low at this cut-off, increasing the cut-off to ≥2.5 would reduce the AUC value to 67.9 and result in a low sensitivity of 51.4%. Chi-square tests were statistically significant for all these cut-off values at p < 0.001.

**Table 2:** Sensitivity and specificity of different MTA cut-off values in four age decades comparing patients with Alzheimer’s disease and patients with subjective cognitive impairment

AD vs. SCI	<65,y (n = 106)		AUC	65–74,y (n = 186)		AUC	75–84,y (n = 521)		AUC	≥85,y (n = 345)		AUC
	AD n = 18, SCI n = 88			AD n = 95, SCI n = 91			AD n = 399, SCI n = 122			AD n = 313, SCI n = 32		
	SN	SP		SN	SP		SN	SP		SN	SP	
MTA ≥1	**83.3**	**86.4**	84.8	94.7	61.5	78.1	97.5	26.2	61.9	99.7	12.5	56.1
MTA ≥1.5	44.4	96.6	70.5	**73.7**	**84.6**	79.1	84.0	71.3	77.6	89.8	53.1	71.5
MTA ≥2	22.2	98.9	60.5	57.9	91.2	74.6	**73.7**	**76.2**	75.0	**84.0**	**62.5**	73.3
MTA ≥2.5	5.6	98.9	52.2	24.2	98.8	61.6	43.4	93.4	68.4	51.4	84.4	67.9
MTA ≥3	0.0	100.0	50.0	11.6	98.9	55.2	27.3	97.5	62.4	38.0	87.5	62.8
MTA ≥3.5	0.0	100.0	50.0	2.1	100.0	51.1	6.5	99.2	52.8	11.8	100.0	55.9
MTA ≥4	0.0	100.0	50.0	2.1	100.0	51.1	3.0	100.0	51.5	4.5	100.0	52.2

Sensitivity (SN) and specificity (SP) values and area under the curve (AUC, computed with receiver operator characteristic curves) for medial temporal atrophy (MTA) visual rating scale (Scheltens) on computed tomography as marker for patients with Alzheimer’s disease (AD) compared to patients with subjective cognitive impairment (SCI). MTA (mean of right and left value) is considered positive when MTA is higher or equal to the indicated cut-off value. For example at MTA ≥1 under 65 years: 83.3% of AD patients and 13.6% of SCI patients had MTA ≥1. Optimal combinations of sensitivity and specificity are in bold

ICC values (95% CI) for intra-rater reliability were 0.98 (0.96–0.99) for the first team of neurologist and geriatrician, 0.97 (0.94–0.99) for the second team, and 0.91 (0.79–0.96) for the neuroradiologist, demonstrating good (ICC value >0.70) to very good reliability and thus consistent ratings over time. The ICC value for inter-rater reliability between the two teams of neurologist and geriatrician was 0.95 (0.89–0.98) showing good reliability between the two teams. ICC value for inter-rater reliability between the first team of neurologist and geriatrician and the neuroradiologist was 0.88 (0.73–0.95), and 0.87 (0.71–0.95) for the second team and the neuroradiologist. These results show good reliability between the teams and the neuroradiologist.

## Discussion

We found that MTA strongly increased with age, but that the age effect is much stronger below 80 years than above, at the same rate in both AD patients and patients with SCI. In addition, optimal MTA cut-off scores to differentiate AD from SCI were ≥1.0, ≥1.5, ≥2.0 and ≥2.0 for the four respective decades.

Increase of MTA with age showed similar patterns in AD and controls [18, 19] and atrophy in these groups showed overlap especially in the hippocampus with increasing age [20]. However, few subjects over 80 years old are studied [21], and whether the slopes of the regression lines of age and MTA diverge, converge or continue to remain parallel between AD and controls was unknown in these elderly persons. We now demonstrate that the effect of age on MTA is similar in both AD and SCI, even at advanced age, and that this effect attenuates after 80 years, again to a similar degree. This may represent a ceiling effect of the visual MTA rating scale or a true absence of patients with severe hippocampal atrophy in our population, possibly explained by selective referral. When differences between groups remain largely the same even at high age, MTA may retain its diagnostic capacity to differentiate AD from control subjects or SCI patients in a memory clinic setting in these very elderly individuals but further analyses in this study do not support this notion.

MTA visual rating was proposed by Scheltens et al. in 1992 [7] and since then this scale has been used in research studies, validated for AD patients [8], incorporated in clinical criteria [5], modified by several authors [22–24], and shown to be reproducible among observers [25]. The use of the Scheltens scale in routine clinical practice is still limited, however, in part due to lack of validation [26]. The average of right and left MTA score is suggested as the best marker [12] and recommendations were made for the use of 1.5 under 75 years and 2 or more for those over 75 years as cut-off scores [11]. Only recently has the approach been taken to compute decade-specific MTA cut-off scores optimize sensitivity and specificity [13], and these cut-off scores were used in a recent study on quantitative electroencephalography in addition to dementia biomarkers [27]. We adopted this strategy and tested the results in a memory clinic setting and found comparable results for the middle-age ranges, despite methodological differences including different AD source population (single-centre memory clinic vs. academic centres and private practice), different reference population (patients with SCI versus normal controls), different rating procedure (consensus rating vs. one single rater) and different imaging tool (CT vs. MRI). With the exception of the cut-off scores under 65 years and above 85 years of age [13], MTA decade-specific cut-off values of 1.5 and 2.0 were the same in the age ranges 65–74 years and 75–84 years. Our study is the first to use a large unselected population of AD patients in one clinical centre demonstrating that a decade-specific adjustment is needed for MTA cut-off scores.

Optimal combinations of sensitivity and specificity are based on the highest value of AUC in studies, for example in Ferreira et al. [13]. This may not always be the best approach to define the clinical usefulness of MTA as an instrument to support the diagnosis of AD. As clinicians, we must keep the chance of making a false-positive diagnosis using MTA as low as possible. In our study it is apparent that in the age group of 75–84 years the cut-off with the highest AUC value (77.6) has a high sensitivity of 84.0% but the corresponding value of specificity is lower and false positive rates fall close to 30% (MTA ≥ 1.5 in 75–84 years). Therefore, from a clinical perspective, we increased the cut-off from 1.5 to 2.0 to increase specificity and accept a lower sensitivity. AUC values from the cut-offs 1.5 and 2.0 are very comparable, 77.6 versus 75.0.

Our finding that the age effect on MTA decelerates after 80 years would suggest not increasing the cut-off after 85 years. Based on the AUC values, this is precisely what our results suggest with the highest AUC value being 73.3 at cut-off ≥ 2.0 and corresponding high sensitivity of 84.0% but low specificity of 62.5%. Increasing the cut-off value to ≥ 2.5 for above 85 years, however, results in our opinion in an unacceptably low sensitivity value of 51.4%. Therefore, above 85 years, there is either low specificity (cut-off ≥2.0) or low sensitivity (cut-off ≥2.5), limiting the practical use of MTA above the age of 85 years. We suggest using the MTA cut-off ≥2.0 above the age of 85 years, bearing in mind that there is the risk of false positives. However, using the data from Table 2, clinicians may decide themselves how to use the MTA cut-off scores, e.g. using presence of MTA ≥2.5 for over 85 years as highly suggestive for AD with a 16% chance of false positives.

AUC values of our study are generally somewhat lower than the Ferreira study [13], with a small age effect, with largest differences in the highest age groups (decade-specific differences 2.9, 1.7, 4.9 and 5.9 respectively). Since AUC is a combined measure of sensitivity and specificity, this may be due to either of these values. It appears that it is not the specificity that is responsible for this difference, with even higher specificity values in our study in the suggested cut-off scores than the Ferreira study, with the exception of the 2.5 cut-off. Interestingly, this suggests that our SCI group is very much comparable to the normal control group in the Ferreira study. In our study sensitivity is lower in all age groups, being constantly 10% lower in all four age groups. Thus the percentage of AD patients having MTA scores above the cut-off scores is about 10% higher in the Ferreira study [13], probably explained by our unselected AD patients referred for evaluation to a general memory clinic.

Methodological considerations include our use of SCI as reference group, opposed to the use of normal controls in other studies. The SCI group may contain patients at risk for cognitive decline or dementia [28] and many may fulfill criteria for subjective cognitive decline [29]. This may result in underestimating the difference between groups. Although this possibility cannot be excluded, it seems unlikely since comparison of our SCI patients with the normal controls in the Ferreira study [13] shows that similarities outweigh differences with a slightly lower MMSE score but a lower MTA score in SCI. Moreover, the use of SCI as a reference group may increase face validity and be more advantageous, since our study confirms that within the group of referred elderly patients to a memory clinic, differentiation can be made between AD and SCI. A mean MMSE score of 18.0 clearly shows that this study represents the whole range of severity in a memory clinic population.

Our MTA visual rating procedure is different from previous studies. Some of these studies employ a single rater with much experience in the field [12, 13, 30]; however, this procedure is not easily translated to the general clinical situation. The advantage of a single rater is high intra-rater reliability as opposed to the risk of higher variability with different raters or with consensus ratings. There is an effect of expertise and practice in the visual rating of MTA when expert neuro-radiologists are compared to non-expert readers [31]. But, as the authors emphasize, in general clinical practice ratings are often performed by radiologists with less experience, as is the case in our hospital. Therefore, since expertise plays a crucial role in MTA ratings, the radiological reports could not be used as gold standard and we employed a consensus rating by an experienced neurologist and geriatrician. This rating procedure may be more representative for usual clinical practice and further supports the generalizability of our findings to comparable clinical settings.

Another reason for consensus rating is to decrease inter-individual variability. Our intra-rater variability study shows reliable and consistent ratings over time for the two teams of neurologist and geriatrician and for the neuroradiologist. Also the inter-rater studies show good reliability between all raters, further adding to the validity of our results. Our study suggests practical usefulness of MTA and may give impetus to more routine use of MTA ratings by radiologists and increase experience in these assessments.

Comparison of visual rating and volumetric measurement of MTA on MRI has received much investigative attention. While strenuous efforts are made to develop standardized protocols for manual segmentation in hippocampal volumetry on MRI [32, 33], there is a need to test visual rating procedures, due to its easier use in clinical practice. Indeed, the most frequently used biomarker is visually rated MTA [34]. In distinguishing AD patients versus normal controls several studies now indicate that visual rating of MTA on MRI is equivalent to volumetric measurement [28, 29, 35–37]. Although MRI has higher resolution and provides no radiation exposure, we used CT in our study as it was earlier suggested that CT imaging may be equivalent for visual assessment compared to MRI when a 64-slice CT is used in a practical clinical situation [17]. Another consideration was that in the elderly population CT is more easily applicable and convenient for patients. Our findings thus suggest that CT may serve as an equivalent imaging tool as MRI in these elderly individuals for this purpose. This may have important consequences for clinical practice in memory clinics.

Our study has several limitations. We have no CSF support for our AD diagnosis and pathological confirmation is not available. Thus, diagnostic misclassification may play a role and overestimation of MTA in the differentiation between SCI and AD cannot be excluded. Indeed, very old patients with moderate to severe MTA may be misdiagnosed since this may not always reflect Alzheimer-type pathology [38]. Further studies are needed in terms of test-retest studies, correlation with clinical measures [26] and post-mortem studies [39]. Ferreira et al. suggest that carrying the ApoE ε4 allele may have an impact on MTA values, showing that ApoE ε4 carriers had higher MTA scores, but only in those under 65 years of age [13]. We did not have ApoE genotyping available. Furthermore, there is the risk that MTA assessments played a role in making the diagnosis of SCI or AD in our study. However, MTA ratings were not part of the diagnostic process, partly because reliable cut-off values were not available. Finally, we did not report MTA in relation to mild cognitive impairment or other dementia diagnoses, such as fronto-temporal dementia, dementia with Lewy bodies or dementia with Parkinson’s disease, as this was not the focus of current study. However, this scope is important in clinical practice and should receive future investigative attention.

In conclusion, we suggest decade-specific MTA cut-off scores for Alzheimer’s disease in the elderly. Visual MTA assessment using CT scans is feasible with high face validity in a memory clinic setting and these cut-off scores may now be adopted in routine clinical practice.

## References

[1] Frisoni GB, Fox NC, Jack CR (2010). The clinical use of structural MRI in Alzheimer disease. Nat Rev Neurol.

[2] Whitwell JL, Jack CR, Przybelski SA (2011). Temporoparietal atrophy: a marker of AD pathology independent of clinical diagnosis. Neurobiol Aging.

[3] McKhann GM, Knopman DS, Chertkow H (2011). The diagnosis of dementia due to Alzheimer’s disease: recommendations from the National Institute on Aging-Alzheimer’s Association workgroups on diagnostic guidelines for Alzheimer’s disease. Alzheimers Dement.

[4] Albert MS, DeKosky ST, Dickson D (2011). The diagnosis of mild cognitive impairment due to Alzheimer’s disease: recommendations from the National Institute on Aging-Alzheimer’s Association workgroups on diagnostic guidelines for Alzheimer’s disease. Alzheimers Dement.

[5] Dubois B, Feldman HH, Jacova C (2007). Research criteria for the diagnosis of Alzheimer’s disease: revising the NINCDS–ADRDA criteria. Lancet Neurol.

[6] Korf ESC, Wahlund L-O, Visser PJ (2004). Medial temporal lobe atrophy on MRI predicts dementia in patients with mild cognitive impairment. Neurology.

[7] Scheltens P, Leys D, Barkhof F (1992). Atrophy of medial temporal lobes on MRI in ‘probable’ Alzheimer’s disease and normal ageing: diagnostic value and neuropsychological correlates. J Neurol Neurosurg Psychiatry.

[8] Scheltens P, van de Pol L (2012). Atrophy of medial temporal lobes on MRI in “probable” Alzheimer’s disease and normal ageing: diagnostic value and neuropsychological correlates. J Neurol Neurosurg Psychiatry.

[9] Tam CWC, Burton EJ, McKeith IG (2005). Temporal lobe atrophy on MRI in Parkinson disease with dementia: a comparison with Alzheimer disease and dementia with Lewy bodies. Neurology.

[10] Galton CJ, Patterson K, Graham K (2001). Differing patterns of temporal atrophy in Alzheimer’s disease and semantic dementia. Neurology.

[11] Van de Pol LA, Scheltens P (2014). Medial temporal lobe atrophy scores translated to clinical practice: editorial comment on ‘Influence of age, disease onset and ApoE4 on visual medial temporal lobe atrophy cut-offs’. J Intern Med.

[12] Pereira JB, Cavallin L, Spulber G (2014). Influence of age, disease onset and ApoE4 on visual medial temporal lobe atrophy cut-offs. J Intern Med.

[13] Ferreira D, Cavallin L, Larsson E-M (2015). Practical cut-offs for visual rating scales of medial temporal, frontal and posterior atrophy in Alzheimer’s disease and mild cognitive impairment. J Intern Med.

[14] Walhovd KB, Fjell AM, Reinvang I (2005). Effects of age on volumes of cortex, white matter and subcortical structures. Neurobiol Aging.

[15] Van der Flier WM, Pijnenburg YA, Prins et al (2014) Optimizing patient care and research: the Amsterdam Dementia Cohort. J Alzheimers Dis 313–27 10.3233/JAD-132306 24614907

[16] Claus JJ, Staekenborg SS, Roorda JJ (2016). Low prevalence of mixed dementia in a cohort of 2,000 elderly patients in a memory clinic setting. J Alzheimers Dis.

[17] Wattjes MP, Henneman WJ, van der Flier WM (2009). Diagnostic imaging of patients in a memory clinic: comparison of MR imaging and 64-detector row CT. Radiology.

[18] Van de Pol LA, Hensel A, Barkhof F (2006). Hippocampal atrophy in Alzheimer disease: age matters. Neurology.

[19] Jack CR, Petersen RC, Xu Y (2000). Rates of hippocampal atrophy correlate with change in clinical status in aging and AD. Neurology.

[20] Raji CA, Lopez OL, Kuller LH (2009). Age, Alzheimer disease, and brain structure. Neurology.

[21] Bakkour A, Morris JC, Wolk DA (2013). The effects of aging and Alzheimer’s disease on cerebral cortical anatomy: specificity and differential relationships with cognition. Neuroimage.

[22] Galton CJ, Gomez-Anson B, Antoun N (2001). Temporal lobe rating scale: application to Alzheimer's disease and frontotemporal dementia. J Neurol Neurosurg Psych.

[23] Duara R, Loewenstein DA, Potter E (2008). Medial temporal lobe atrophy on MRI scans and the diagnosis of Alzheimer disease. Neurology.

[24] Kaneko T, Kaneko K, Matsushita M (2012). New visual rating system for medial temporal lobe atrophy: a simple diagnostic tool for routine examinations. Psychogeriatrics.

[25] Sarria-Estrada S, Acevedo C, Mitjana R (2015). Reproducibility of qualitative assessments of temporal lobe atrophy in MRI studies. Radiologia.

[26] Harper L, Barkhof F, Fox NC (2015). Using visual rating to diagnose dementia: a critical evaluation of MRI atrophy scales. J Neurol Neurosurg Psychiatry.

[27] Ferreira D, Jelic V, Cavallin L (2016). Electroencephalography is a good complement to currently established dementia biomarkers. Dement Geriatr Cogn Disord.

[28] Slavin MJ, Sachdev PS, Kochan NA (2015). Predicting cognitive, functional, and diagnostic change over 4 years using baseline subjective cognitive complaints in the Sydney Memory and Ageing Study. J Geriatr Psychiatry Neurol.

[29] Jessen F, Amariglio RE, van Boxtel M (2014). A conceptual framework for reasearch on subjective cognitive decline in preclinical Alzheimer's diease. Alzheimers Dement.

[30] Westman E, Cavallin L, Muehlboeck J-S (2011). Sensitivity and specificity of medial temporal lobe visual ratings and multivariate regional MRI classification in Alzheimer’s disease. PLoS One.

[31] Boutet C, Chupin M, Colliot O (2012). Is radiological evaluation as good as computer-based volumetry to assess hippocampal atrophy in Alzheimer’s disease?. Neuroradiology.

[32] Boccardi M, Bocchetta M, Apostolova LG (2015). Delphi definition of the EADC-ADNI harmonized protocol for hippocampal segmentation on magnetic resonance. Alzheimers Dement.

[33] Boccardi M, Bocchetta M, Ganzola R (2015). Operationalizing protocol differences for EADC-ADNI manual hippocampal segmentation. Alzheimers Dement.

[34] Bocchetta M, Boccardi M, Ganzola R (2015). Harmonized benchmark labels of the hippocampus on magnetic resonance: the EADC-ADNI project. Alzheimers Dement.

[35] Varon D, Barker W, Loewenstein D (2015). Visual rating and volumetric measurement of medial temporal atrophy in the Alzheimer’s Disease Neuroimaging Initiative (ADNI) cohort: baseline diagnosis and the prediction of MCI outcome. Int J Geriatr Psychiatry.

[36] Duara R, Loewenstein DA, Shen Q (2013). The utility of age-specific cut-offs for visual rating of medial temporal atrophy in classifying Alzheimer’s disease, MCI and cognitively normal elderly subjects. Front Aging Neurosci.

[37] Shen Q, Loewenstein DA, Potter E (2011). Volumetic and visual rating of MRI scans in the diagnosis of amnestic MCI and Alzheimer’s disease. Alzheimers Dement.

[38] Barkhof F, Polvikoski TM, van Straaten ECW (2007). The significance of medial temporal lobe atrophy: a postmortem MRI study in the very old. Neurology.

[39] Harper L, Fumagalli GG, Barkhof F (2016). MRI visual rating scales in the diagnosis of dementia: evaluation in 184 post-mortem confirmed cases. Brain.

